# The effect of L-carnitine supplementation on lipid profile in adults: an umbrella meta-analysis on interventional meta-analyses

**DOI:** 10.3389/fnut.2023.1214734

**Published:** 2023-09-04

**Authors:** Vali Musazadeh, Hanie Alinejad, Niloofar Kouhi Esfahani, Zeynab Kavyani, Majid Keramati, Neda Roshanravan, Erfan Mosharkesh, Parvin Dehghan

**Affiliations:** ^1^Student Research Committee, Tabriz University of Medical Sciences, Tabriz, Iran; ^2^School of Nutrition and Food Sciences, Tabriz University of Medical Sciences, Tabriz, Iran; ^3^Faculty of Pharmacy, Comenius University, Bratislava, Slovakia; ^4^Faculty of Medicine, Rudn University of Moscow, Moscow, Russia; ^5^Cardiovascular Research Center, Tabriz University of Medical Sciences, Tabriz, Iran; ^6^Faculty of Veterinary Medicine, University of Tabriz, Tabriz, Iran; ^7^Nutrition Research Center, Faculty of Nutrition and Food Science, Tabriz University of Medical Sciences, Tabriz, Iran

**Keywords:** L-Carnitine, lipid profile, dyslipidemia, umbrella meta-analysis, systematic reviews

## Abstract

**Introduction:**

Previous meta-analyses investigating the therapeutic effects of L-carnitine on lipid profiles have demonstrated inconsistent results. The present umbrella meta-analysis aimed to investigate the impact of efficacy of L-carnitine on lipid profiles in adults.

**Methods:**

Databases including PubMed, Scopus, and Embase, Web of Science, and Google Scholar were searched up to June 2023. Meta-analysis was performed using a random-effects model.

**Results:**

Our results from thirteen meta-analyses indicated that L-carnitine supplementation significantly total cholesterol (TC) (ES = −1.05 mg/dL, 95% CI: −1.71, −0.39; *p* = 0.002), triglycerides (TG) (ES = −2.51 mg/dL; 95% CI: −3.62, −1.39, *p* < 0.001), and low-density lipoprotein-cholesterol (LDL-C) (ES = −4.81 mg/dL; 95% CI: −6.04, −3.59; p < 0.001). It also increased high-density lipoprotein-cholesterol (HDL-C) (ES: 0.66 mg/dL, 95% CI: 0.20, 1.12, *p* = 0.005) levels.

**Conclusion:**

The present umbrella meta-analysis suggests supplementation with L-carnitine in a dosage of more than 2 g/day can improve lipid profile. Thus, L-carnitine supplementation can be recommended as an adjuvant anti-hyperlipidemic agent.

## Introduction

Chronic heart disease (CHD) is a serious problem in public health in the world. The prevalence of this disease has enhanced considerably in developed and developing countries (1). CHD is a leading cause of death in the world, claiming the lives of up to 17.5 million people each year (2). The underlying cause of CHD is atherosclerosis, an inactive, progressive condition characterized by the deposition of excess cholesterol in the sub endothelial space (3, 4). Dyslipidemia, as one of the most important modifiable risk factors for atherosclerosis, is determined by a disturbance in circulating amounts of triglycerides (TG), total cholesterol (TC), high-density lipoprotein-cholesterol (HDL-C), and low-density lipoprotein-cholesterol (LDL-C) levels (5, 6). Therefore, dyslipidemia is associated with atherosclerosis leading to CHD. Statins are pharmacological drugs used to prevent CHD by reducing plasma LDL-C levels; however, statins do not significantly alter other lipid indices (7, 8). Also, these common medications can cause poisoning and side effects such as myotoxicity, intracranial hemorrhage, and coenzyme Q10 deficiency in patients (3, 4, 9). Currently, natural compounds are used for the prevention and treatment of chronic diseases, and the use of natural compounds with antioxidant, anti-inflammatory, lipid profile modulating, and blood pressure lowering properties as oral supplements are one of the new ways to prevent and treat chronic diseases (10, 11).

L-carnitine is an ammonium cation, either obtained from dietary or synthesized in the liver, kidney, and brain. Animal sources like fish, meat, milk, and poultry are the best sources of L-carnitine (12). L-carnitine is necessary for importing long-chain fatty acids (LCFA) into the mitochondrial matrix for beta-oxidation (13) and depleting the acyl groups out of the mitochondria in all tissues (14). Furthermore, L-carnitine can improve adipokines concentrations (15) and decrease the repletion of detrimental metabolites generated in coronary embolism and thrombosis (16–18). Overall, these mechanisms may be improved lipid profile and prevent related diseases.

Several meta-analyses have been conducted to investigate the therapeutic effects of L-carnitine on lipid profiles (19, 20); nevertheless, the results are still inconsistent (21–23). Thus, the present umbrella meta-analysis study was conducted to impart accurate and deterministic data regarding supplementation with L-carnitine on lipid profiles including TG, TC, LDL-C, and HDL-C levels.

### Methods

We used the Preferred Reporting Items for Systematic Reviews and Meta-Analyses (PRISMA) guidelines in this study to analyze the articles (PROSPERO registration number: CRD42022307425) (24).

### Search strategy

To find the relevant studies, five electronic databases, including Scopus, Web of Science, PubMed, Embase and Google Scholar were searched systematically up to June 2023. Search strings were relevant to the L-carnitine on lipid profiles (Supplementary file). In addition, we used the sign of “*” to improve the search literature sensitivity. Besides, a manual search of the references of qualified studies was performed to minimize the risk of missing relevant papers.

### Study selection

Following are the PICOTS criteria: Population/Patients (P: adults of 18 > years of age); Intervention (I: L-carnitine); Comparison (C: control or placebo group); Outcome (O: lipid profile) (TG, HDL-C, LDL-C, and TC); Time (T: studies with a duration of follow-up of ≥2 weeks); and Study design (S: Meta-analyses of RCTs). We included meta-analysis studies examining the impacts of L-carnitine supplementation on lipid profile with their effect sizes (ES) and their corresponding confidence intervals (CI). In addition, other typologies of research studies including *in vivo*, *in vitro* and *ex-vivo* studies, observational studies, case reports, meta-analyses of non-randomized controlled trials or non-controlled trials, and quasi-experimental studies were excluded from the present study.

### Methodological quality and quality of evidence

The methodological quality was evaluated using the Assessing the Methodological Quality of Systematic Reviews (AMSTAR) tool (25). The AMSTAR2 checklist was categorized into “critically low quality,” “low quality,” “moderate quality,” and “high quality.” We evaluate the overall strength and quality of evidence using GRADE according to the Cochrane Handbook of systematic reviews of interventions and based on five factors: precision, consistency of results, bias risk, publication bias, and directness, and. The quality of a level decreases when one of the above factors is not met (26).

### Data extraction

Data that were extracted included the outcomes (ESs and CIs of TG, HDL-C, LDL-C, and TC), information regarding the year of publication, the study’s first author, number of placebo and intervention groups, study location, sample sizes, supplement dosage, and duration.

### Statistical analysis

To evaluate the combined ES of the intervention, ESs, and CIs for lipid parameters were used. Cochran-Q test and *I^2^* index were applied to assess the heterogeneity of the meta-analysis. Significant heterogeneity of data was defined as *I^2^* > 50% or a significant Cochran-Q test (*p* < 0.10). Subgroup analyses were done according to the duration, the dose of L-carnitine, sample size, age, and health status to identify potential sources of heterogeneity. The sensitivity analysis was conducted by the one-study exclude method, to determine the effect of each meta-analysis on the overall ES. Egger’s and Begg’s tests were used to assess the effects of a small study. The funnel plot was evaluated by visual inspection to identify the publication bias. The trim and fill methods were performed if there was a publication bias. All statistical analyses were executed using Stata software (version 16, Stata Corp. College Station, TX, US).

## Results

### Study selection and study characteristics

**Figure 1:**
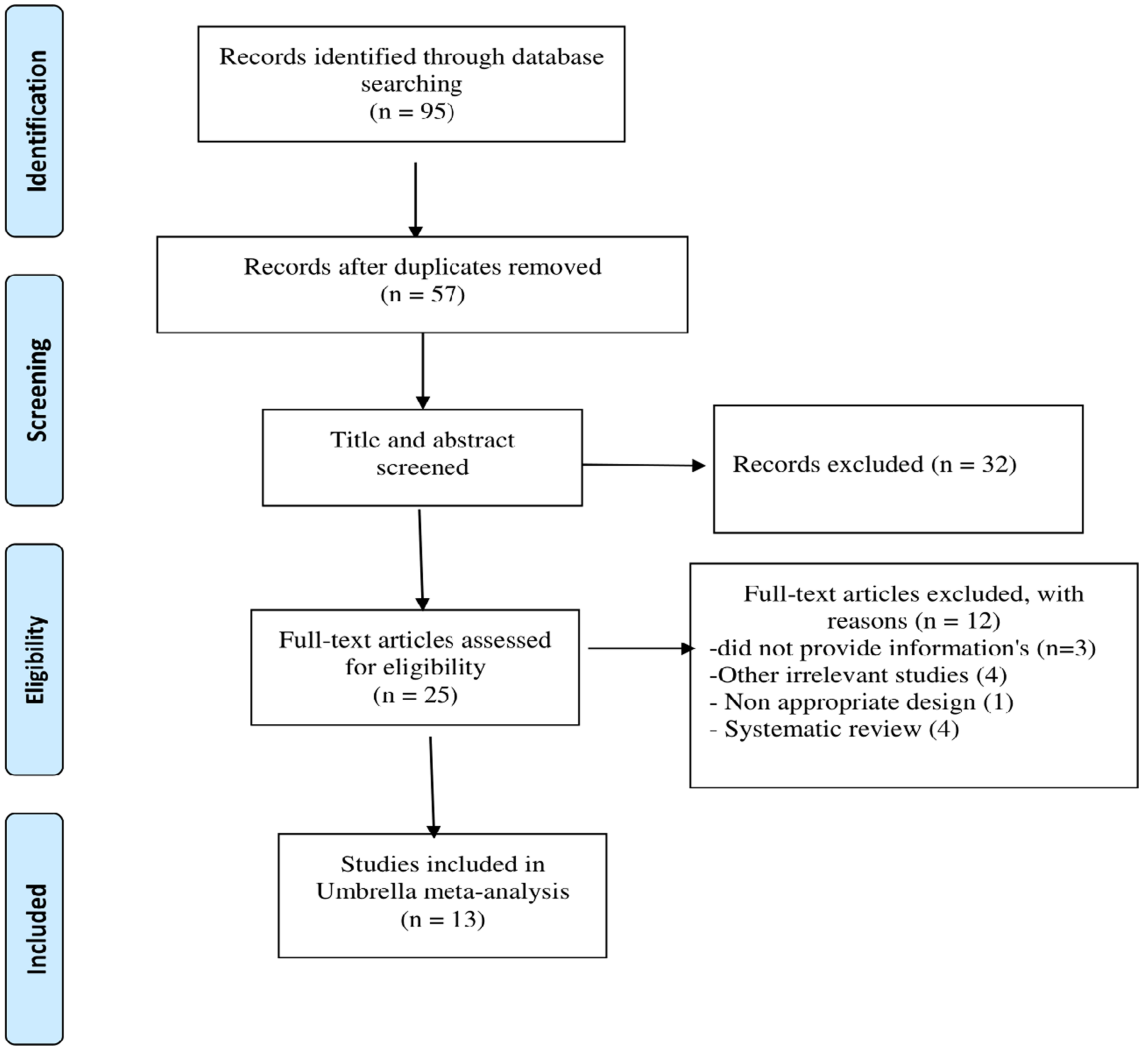
Flow diagram of study selection.

Figure 1 shows the PRISMA flow diagram for the studies. There were 95 articles total after searching electronic databases. After eliminating 38 duplicate articles, 57 papers were carefully evaluated based on titles and abstracts, with 25 being chosen for full-text evaluation. 13 meta-analyses eventually met our inclusion criteria and were included in the present umbrella meta-analysis. The features of the qualified articles are shown in Table 1. The total number of effect sizes identified was 11 for TG, and TC, and 12 for LDL-C, and HDL-C, depending on the type of variable studied. The included studies were published between 2013 and 2021, and the participants’ average age ranged from 26 to 53. L-carnitine administration ranged from 0.54 g/day to 2.4 g/day on average across studies. From 14 to 25 weeks were spent taking L-carnitine supplements Table 1 illustrates the quality of the RCTs incorporated in the current umbrella meta-analysis.

**Table 1:** Study characteristics of included studies.

Citation (First author et al., year)	No. of studies in meta-analysis	Location	no. of participants in meta-analysis	Mean age (year)	dose(g)	Health condition/ duration(wk)	Quality assessment scale and outcome
Casariego et al. 2013	4	Spain	284	NR	2.2	T2DM 25 week	Yes (Jadad) 4/4 high
Abbasnezhad et al. 2020	6	Iran	468	53	1.9	Liver disorders 20 week	Yes (Cochrane) 6/6 high
Abolfathi et al. 2020	4	Malaysia	254	53	0.84	NAFLD 15 week	Yes (Jadad) 4/4 high
Asadi et al. 2020	23	Iran	1,533	NR	1.8	Metabolic disorders 16 week	Yes (Cochrane) 18/23 high
Asbaghi et al. 2020	8	Iran	508	53	2.4	T2DM 19 week	Yes (Cochrane) 8/8 high
Askarpour et al. 2019	56	Iran	3,004	50	1.6	Metabolic disorders 18 week	Yes (Cochrane) NR
Chen et al. 2014	21	China	637	52.5	0.73	ESRD 15 week	Yes (Cochrane) 6/21 high
Choi et al. 2020	6	Korea	375	53	1.6	Metabolic syndrome 14 week	Yes (Cochrane) 4/6 high
Huang et al. 2013	12	China	391	49	0.54	ESRD 18 week	Yes (Jadad) 8/12 high
Liao et al. 2021	3	China	496	26	2.08	PCOS 12 week	Yes (Cochrane) 3/3 high
Yang et al. 2014	11	China	397	50	1.18	ESRD 20 week	Yes (Cochrane) 1/11 high
Gholipur et al.2018	10	Iran	466	NR	1	CKD 21 week	Yes (Cochrane) 5/10 high
Fathizadeh et al. 2019	NR	Iran	NR	NR	NR	NR	NR

### Methodological quality and GRADING-of-evidence

Table 2 shows the results of the quality assessment of qualified studies using the AMSTAR2 questionnaire. Out of 13 studies, ten studies had high-quality and three studies had moderate-quality. Grade assessment revealed low quality for TC and TG, but LDL-c and HDL-c have a moderate, and very low quality of evidence, respectively, (Table 3).

**Table 2:** Results of assess the methodological quality of meta-analysis.

Study	Q 1	Q2	Q3	Q4	Q5	Q6	Q7	Q8	Q9	Q10	Q11	Q12	Q13	Q14	Q15	Q16	Quality assessment
Casariego et al. 2013	No	Partial Yes	Yes	Partial Yes	Yes	Yes	No	Yes	No	No	Yes	No	No	Yes	No	Yes	Moderate
Abbasnezhad et al. 2020	No	Partial Yes	Yes	Partial Yes	Yes	Yes	Partial Yes	Yes	Yes	No	Yes	Yes	Yes	Yes	Yes	Yes	High
Abolfathi et al. 2020	Yes	Yes	Yes	Partial Yes	Yes	Yes	Yes	Yes	Yes	Yes	Yes	Yes	Yes	Yes	Yes	Yes	High
Asadi et al. 2020	No	Yes	Yes	Partial Yes	Yes	Yes	Yes	Yes	Yes	No	Yes	Yes	Yes	Yes	No	Yes	High
Asbaghi et al. 2020	Yes	Yes	Yes	Partial Yes	Yes	Yes	Yes	Yes	Yes	Yes	Yes	Yes	Yes	Yes	Yes	Yes	High
Askarpour et al. 2019	Yes	Partial Yes	Yes	Partial Yes	Yes	Yes	Yes	Yes	Yes	No	Yes	No	Yes	Yes	Yes	Yes	High
Chen et al. 2014	No	Yes	Yes	Partial Yes	Yes	Yes	Partial Yes	Yes	Yes	No	Yes	No	Yes	No	Yes	Yes	Moderate
Choi et al. 2020	No	Yes	Yes	Partial Yes	No	Yes	Yes	Partial Yes	Yes	Yes	Yes	No	Yes	Yes	No	Yes	Moderate
Huang et al. 2013	No	Yes	Yes	Partial Yes	No	No	Yes	Yes	Yes	Yes	Yes	Yes	Yes	Yes	Yes	Yes	Moderate
Liao et al. 2021	No	Yes	Yes	Partial Yes	Yes	Yes	Yes	Yes	Yes	Yes	Yes	Yes	Yes	No	Yes	Yes	High
Yang et al. 2014	No	Yes	Yes	Partial Yes	Yes	Yes	Partial Yes	Yes	Yes	No	Yes	Yes	No	No	Yes	No	Moderate
Gholipur et al.2018	Yes	Yes	Yes	Partial Yes	Yes	Yes	Partial Yes	Yes	Yes	Yes	Yes	Yes	No	No	No	Yes	Moderate
Fathizadeh et al. 2019	No	No	No	No	No	No	No	No	No	No	No	No	No	No	No	No	Critically low

**Table 3:** Summary of results and quality of evidence assessment using the GRADE approach.

Outcome measure	Summary of findings		Quality of evidence assessment (GRADE)					
	No of patients (trials)	Effect size (95% CI)	Risk of bias ^a^	Inconsistency ^b^	Indirectness ^c^	Imprecision ^d^	Publication bias ^e^	Quality of evidence ^f^
Lipid profile								
LDL-C (mg/dl)	6,751 (51)	−4.81 (−6.04, −3.59)	Not Serious	Not Serious	Serious	Not Serious	Not Serious	Moderate
HDL-C (mg/dl)	7,010 (54)	0.66 (0.20, 1.12)	Not Serious	Not Serious	Serious	Serious	Serious	Very Low
TG (mg/dl)	8,075 (59)	−2.51 (−3.62, −1.39)	Not Serious	Not Serious	Serious	Serious	Not Serious	Low
TC (mg/dl)	8,006 (58)	−1.05 (−1.71, −0.39)	Not Serious	Not Serious	Serious	Serious	Not Serious	Low

LDL, low-density lipoprotein; HDL, high-density lipoprotein; TG, triglyceride; TC, total cholesterol.

a Risk of bias based on the AMSTAR2 results.

b Downgraded if there was a substantial unexplained heterogeneity (I2 > 50%, P < 0.10) that was unexplained by meta-regression or subgroup analyses.

c Downgraded if there were factors present relating to the participants, interventions, or outcomes that limited the generalizability of the results.

d Downgraded if the 95% confidence interval (95% CI) crossed the minimally important difference (MID) for benefit or harm. MIDs used for each outcome were: 3.87 mg/dL for LDL, HDL, and TC, 8.86 mg/dL for TG.

e Downgraded if there was an evidence of publication bias using funnel plot.

f Since all included studies were meta-analyses of randomized control trials, the certainty of the evidence was graded as high for all outcomes by default and then downgraded based on prespecified criteria. Quality was graded as high, moderate, low, very low.

### L-carnitine on TC

**Figure 2:**
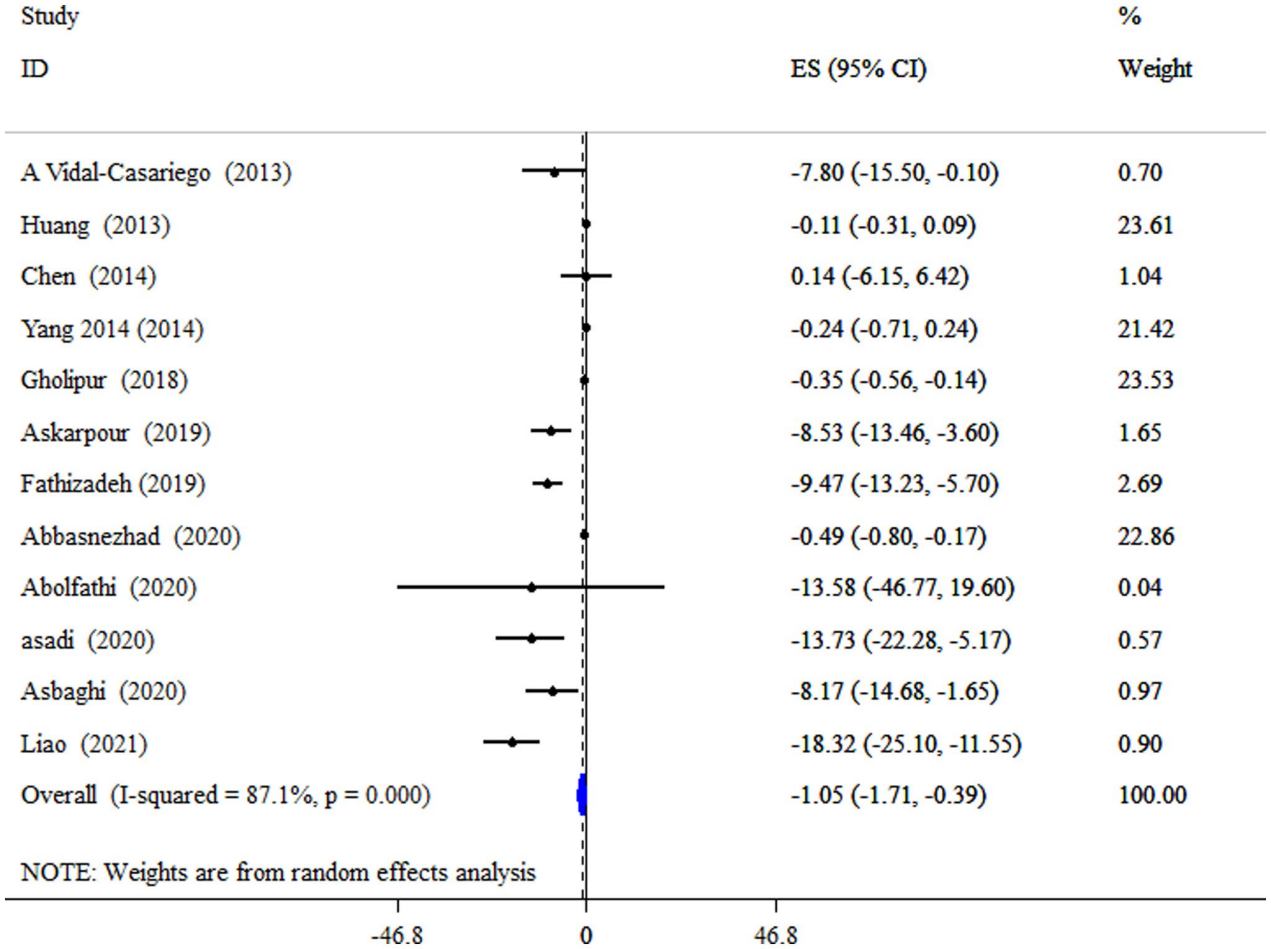
Forest plot detailing mean difference and 95% confidence intervals (CIs), the impacts of L-carnitine supplementation on TC levels.

L-carnitine supplementation had a significant lowering effect on TC level (ES = −1.05 mg/dL, 95% CI: −1.71, −0.39; *p* = 0.002), with a significant between-study heterogeneity (*I^2^* = 87.1%, *p* < 0.001) (Figure 2). In subjects with a mean age of under 50 years old and a supplement of L-carnitine >2 g/day resulted in a notable reduction of TC (Table 4).

**Table 4:** Subgroup analyses for the effects of L-Carnitine supplementation on lipid profile.

	Effect size, *n*	ES (95% CI)^1^	P-within^2^	*I*^2^ (%)^3^	P-heterogeneity^4^
L-Carnitine on TC levels					
Overall	12	−1.05 (−1.71, −0.39)	0.002	87.1	<0.001
Age(year)					
≤50	4	−1.56 (−3.02, −0.09)	0.037	92.3	<0.001
>50	4	−2.23 (−6.23, 1.77)	0.275	49.7	0.113
NR	4	−7.25 (−14.19, −0.31)	0.041	91.5	<0.001
Intervention duration (week)					
<18	3	−16.48 (−21.72, −11.23)	<0.001	0.0	0.701
≥18	8	−0.38 (−0.74, −0.02)	0.040	72.0	<0.001
NR	1	−9.47 (−13.23, −5.70)	<0.001	–	–
Study population					
T2DM	2	−8.02 (−12.99, −3.04)	0.002	0.0	0.943
Metabolic disorders	2	−9.91 (−14.40, −5.41)	<0.001	6.1	0.302
CKD	4	−0.22 (−0.36, −0.08)	0.002	0.0	0.459
NAFLD	1	−13.58 (−46.76, 19.60)	0.423	–	–
Liver disorders	1	−0.49 (−0.81, −0.18)	0.002	–	–
PCOS	1	−18.32 (−25.10, −11.55)	<0.001	–	–
NR	1	−9.47 (−13.23, −5.70)	<0.001	–	–
Dosage (g/day)					
≤ 2	8	−0.37 (−0.73, 0.00)	0.051	72.9	<0.001
> 2	3	−11.51 (−18.36, −4.66)	<0.001	65.4	0.056
NR	1	−9.47 (−13.23, −5.70)	<0.001	–	–
L-Carnitine on LDL-C levels					
Overall	11	−4.81 (−6.04, −3.59)	<0.001	96.8	<0.001
Age(years)					
≤50	4	−8.79 (−19.73, 2.16)	0.116	98.5	<0.001
>50	3	−3.12 (−7.39, 1.14)	0.151	77.1	0.013
NR	4	−5.68 (−11.14, −0.22)	0.042	97.1	<0.001
Intervention duration (week)					
<18	4	−11.28 (−19.76, −2.81)	0.009	89.9	<0.001
≥18	6	−1.83 (−2.71, −0.95)	<0.001	94.8	<0.001
NR	1	−6.25 (−9.30, −3.20)	<0.001	–	–
Study population					
T2DM	2	−7.56 (−10.90, −4.23)	<0.001	55.2	0.135
Metabolic disorders	2	−6.26 (−8.68, −3.83)	<0.001	0.0	0.392
CKD	3	−0.34 (−0.58, −0.09)	0.007	45.4	0.160
NAFLD	1	−14.85 (−45.43, 15.73)	0.341	–	–
Liver disorders	1	−0.20 (−0.57, 0.17)	0.296	–	–
PCOS	1	−18.91 (−21.58, −16.25)	<0.001	–	–
NR	1	−6.25 (−9.30, −3.20)	<0.001	–	–
Dosage (g/day)					
≤ 2	7	−0.57 (−1.07, −0.07)	0.025	79.1	<0.001
> 2	3	−11.08 (−18.73, −3.43)	0.005	95.6	<0.001
NR	1	−6.25 (−9.30, −3.20)	<0.001		–
L-Carnitine on HDL-C levels					
Overall	11	0.66 (0.20, 1.12)	0.005	72.2	<0.001
Age (years)					
≤50	4	1.18 (−0.38, 2.75)	0.139	78.2	0.003
>50	4	0.12 (−0.25, 0.49)	0.511	5.2	0.367
NR	3	0.89 (0.52, 1.25)	<0.001	0.0	0.546
Intervention duration (week)					
<18	5	1.19 (0.21, 2.17)	0.017	26.9	0.242
≥18	5	0.23 (−0.18, 0.65)	0.272	57.6	0.051
NR	1	1.39 (0.21, 2.57)	0.021	–	–
Study population					
T2DM	2	0.36 (−1.91, 2.63)	0.754	38.6	0.202
Metabolic disorders	3	1.04 (0.51, 1.57)	<0.001	18.3	0.294
CKD	2	0.02 (−0.35, 0.40)	0.907	0.0	0.541
NAFLD	1	1.36 (−0.96, 3.68)	0.251	–	–
Liver disorders	1	0.08 (−0.02, 0.17)	0.099	–	–
PCOS	1	10.27 (1.67, 18.88)	0.019	–	–
NR	1	1.39 (0.21,2.57)	0.021	–	–
Dosage (g/day)					
≤ 2	7	0.54 (0.09, 1.00)	0.018	75.7	<0.001
> 2	3	2.00 (−1.87, 5.87)	0.310	70.4	<0.001
NR	1	1.39 (0.21, 2.57)	0.021	–	–
L-Carnitine on TG levels					
Overall	12	−2.51 (−3.62, −1.39)	<0.001	92.8	<0.001
Age (years)					
≤50	4	−4.25 (−6.48, −2.03)	<0.001	97.7	<0.001
>50	5	−5.59 (−14.45, 3.27)	0.216	61.5	0.034
NR	3	−7.69(−12.23, −3.15)	<0.001	0.0	0.388
Intervention duration (week)					
<18	5	−13.22 (−18.12, −8.32)	<0.001	21.5	0.278
≥18	6	−0.15 (−0.42, 0.12)	0.277	46.0	0.099
NR	1	−10.35 (−16.43, −4.27)	<0.001	–	–
Study population					
T2DM	2	−0.05 (−7.45, 7.36)	0.990	0.0	0.484
Metabolic disorders	3	−9.00 (−14.23, −3.76)	<0.001	0.0	0.473
CKD	3	−0.09 (−0.29, 0.12)	0.421	0.0	0.850
NAFLD	1	−14.51 (−17.06, −11.97)	<0.001	–	–
Liver disorders	1	−22.13 (−38.92, −5.34)	0.010	–	–
PCOS	1	−0.23 (−0.41, −0.05)	0.012	–	–
NR	1	−10.35 (−16.43, −4.27)	<0.001	–	–
Dosage (g/day)					
≤ 2	8	−0.19 (−0.61, 0.23)	0.371	65.7	0.005
> 2	3	−5.64 (−17.40, 6.12)	0.347	85.3	<0.001
NR	1	−10.35 (−16.43, −4.27)	<0.001	–	–

ES, effect size; CI, confidence interval; 1Obtained from the Random-effects model, 2Refers to the mean (95% CI), 3Inconsistency, percentage of variation across studies due to heterogeneity, 4Obtained from the Q-test, NR, Not reported; T2DM, Type 2 diabetes mellitus; MetS, Metabolic syndrome, CKD, Chronic kidney disease; NAFLD, Non-alcoholic fatty liver disease; PCOS, Polycystic Ovary Syndrome.

### L-carnitine on TG

**Figure 3:**
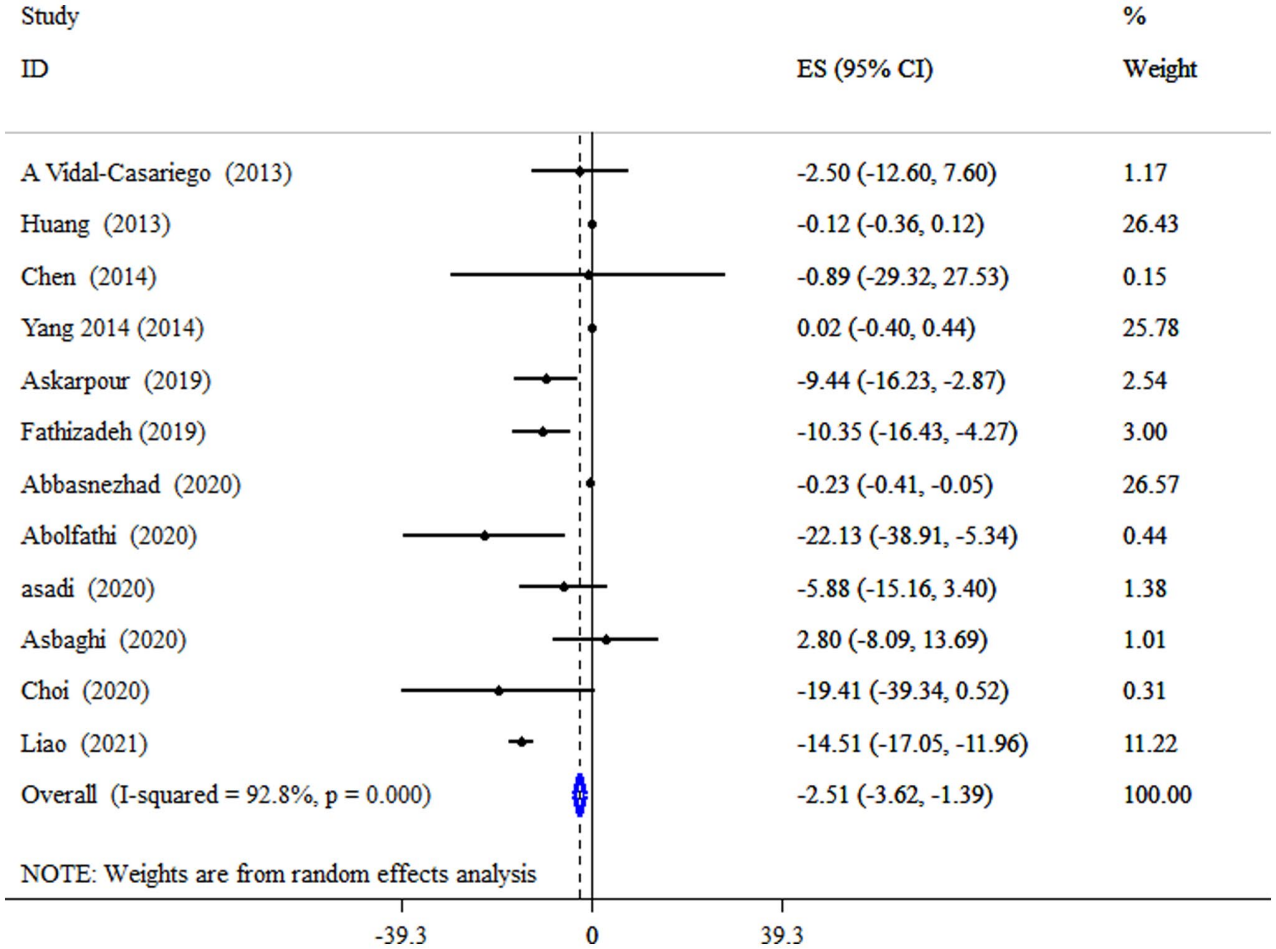
Forest plot detailing mean difference and 95% confidence intervals (CIs), the impacts of L-carnitine supplementation on TG levels.

According to the pooled estimate, subjects who took supplements of L-carnitine had significantly decreased levels of TG (ES = −2.51 mg/dL; 95% CI: −3.62, −1.39, *p* < 0.001;12 meta-analyses) (Figure 3). The studies had significant between-study heterogeneity (*I^2^* = 92.8%, *p* < 0.001). Subgroup analysis revealed that L-carnitine supplementation has a more pronounced effect on lowering TG levels in subjects with metabolic disorders who have a mean age of less than 50 years and an intervention duration of less than 18 weeks (Table 4).

### L-carnitine on LDL-C

**Figure 4:**
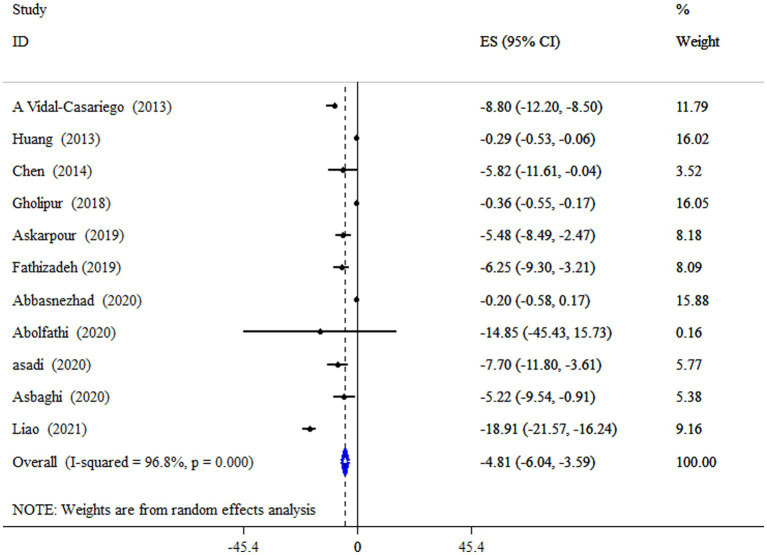
Forest plot detailing mean difference and 95% confidence intervals (CIs), the impacts of L-carnitine supplementation on LDL-C levels.

Data from four meta-analyses indicated that L-carnitine supplementation significantly reduced LDL-C levels (ES = −4.81 mg/dL; 95% CI: −6.04, −3.59; *p* < 0.001;11 meta-analyses) (Figure 4). Studies showed a significant degree of heterogeneity (*I^2^* = 96.8%, *p* < 0.001). In studies with a dosage of more than 2 g/day on subjects with type 2 diabetes mellitus (T2DM), and an intervention duration of less than 18 weeks, subgroup analysis also revealed a strong impact (Table 4).

### L-carnitine on HDL-C

**Figure 5:**
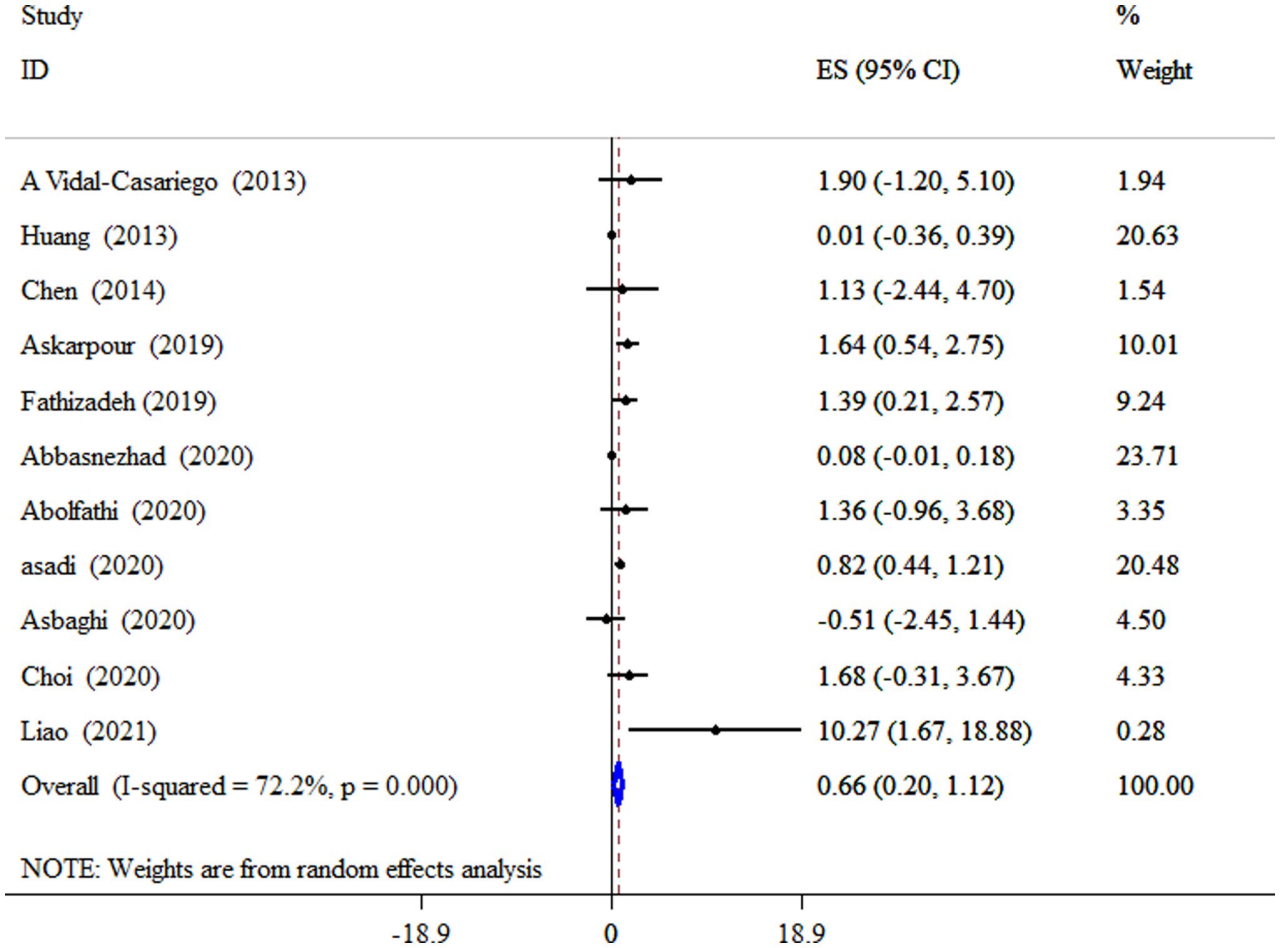
Forest plot detailing mean difference and 95% confidence intervals (CIs), the impacts of L-carnitine supplementation on HDL-C levels.

L-carnitine supplementation significantly increased HDL-C levels (ES = 0.66 mg/dL, 95% CI: 0.20, 1.12, *p* = 0.005; 11 meta-analyses) (Figure 5). Also, a high degree of heterogeneity was detected (*I^2^* = 72.2%, *p* < 0.001). Subgroup analysis revealed that subjects with metabolic disorders who received an 18-week intervention had a more pronounced effect of L-carnitine supplementation on HDL-C levels (Table 4).

### Sensitivity analysis

Sensitivity analysis for TG showed that the elimination of studies by Liao et al. (19) had an effect on the pooled ES, resulting in non-significance (ES = −0.29 mg/dL, 95% CI: −0.81, 0.22; *p* > 0.05). Also, the sensitivity analysis revealed no significance for TC, LDL-C, and HDL-C.

### Publication bias, trim and fill

Egger’s unlike Begg’s test has revealed a significant small-study effect on TC (*p* = 0.001 and 0.193), LDL-C (*p* = 0.011 and 0.999), and HDL-C (*p* = 0.013 and 0.755) levels. In contrast to other parameters, no evidence of a significant small study effect was found for TG (*p* = 0.945 for Begg’s and *p* = 0.055 for Egger’s test). Publication bias was identified through a visual assessment of the funnel plot (Supplementary file). We conducted the trim and fill test since the funnel plot assessment’s visual inspection revealed an uneven distribution of studies for all outcomes. Unlike other parameters, trim and fill analysis was performed on HDL-C level with 16 studies (five imputed studies); thus, correction for potential publication bias altered the effect of L-carnitine on HDL-C (ES = 0.31 mg/dL; 95% CI: −0.13, 0.74, *p* > 0.05).

## Discussion

L-carnitine supplementation could have antihyperlipidemic effects according to our pooled analysis on 13 meta-analyses consisting of 51, 54, 58, and 59 separate clinical trials for LDL-C, HDL-C, TC and TG, respectively. As far as we know, this is the first umbrella meta-analysis of RCTs in the realm of the clinical benefits of L-carnitine supplementation on lipid parameters. Based on the results, TC, TG, LDL-C were significantly decreased after L-carnitine supplementation. Also, results from our study demonstrated that consuming carnitine supplements improved HDL-C levels.

Previous reports have proven that dyslipidemia are independent predictors of cardiovascular disease (CVD) risk. Accumulating evidence has suggested potentially beneficial properties of L-carnitine as nutraceutical for managing dyslipidemia and prevent of CVD. L-carnitine as a non-protein amino acid is the known carrier of fatty acids (FAs) across the inner mitochondrial membrane and plays an important role in the metabolism of FAs and activation of β-oxidation via regulating long-chain FAs transport from the mitochondrial membrane. Endogenous production of L-carnitine can be done in kidneys and liver from lysine, methionine ascorbate, niacin, pyridoxine, and iron (27).

**Figure 6:**
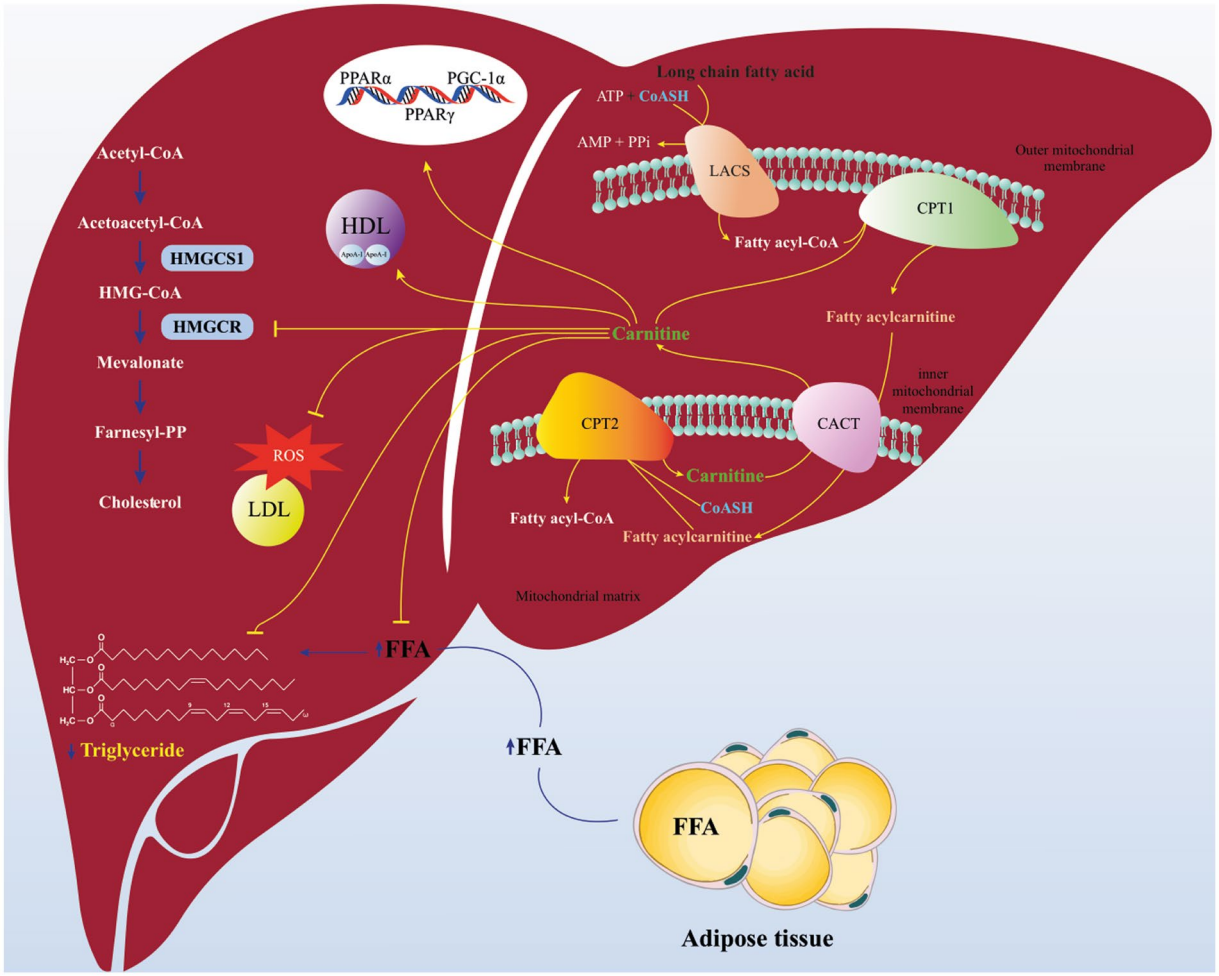
Schematics of proposed pathway for beneficial effects of L-carnitine on lipid parameters. L-carnitine enhanced the mitochondrial oxidation (beta-oxidation) of long Chain-Acyl CoA. Carnitine can reduce the availability of free fatty acids (FFAs), diminish conversion of FFAs to triglycerides. L-carnitine can affect cholesterol synthesis pathway (mevalonate pathway) *via* inhibition of β-hydroxy β-methylglutaryl (HMG)-CoA reductase activity. Carnitine can improve lipid profile by modifying the expression of genes associated with lipid metabolism signaling including peroxisome proliferator activated receptor (PPAR α & γ), and peroxisome proliferator-activated receptor γ coactivator 1α (PGC-1α). Also, carnitine can protect LDLs from oxidative stress and promotes the synthesis of HDL *via* increment in Apo-A1 level. Carnitine Acylcarnitine translocase (CACT); Carnitine palmitoyi transferase II (CPT2); Carnitine palmitoyi transferase I (CPT1); Long Acyl-CoA synthetase (LACS).

Scientific evidences associating L-carnitine and disturbances of glucose and lipid metabolism has been reported recently and have suggested L-carnitine as a potential therapeutic agent for some diseases such as T2DM, non-alcoholic fatty liver disease (NAFLD), end-stage kidney disease (ESKD), atherosclerosis, etc. (28, 29). A large number of studies suggested that L-carnitine consumption is associated for normalizing the blood concentrations of TC, TG and LDL-C (22, 30). As shown in Figure 6 the potential positive effects of L-carnitine on the lipid parameters might be explained by several mechanisms. Considering recently published investigations, carnitine deficiency impairs insulin-sensitivity. L-carnitine enhanced the mitochondrial oxidation (beta-oxidation) of long chain-Acyl CoA, which its accumulation triggers insulin resistance in muscle cells and hepatocytes (31). Beyond that, L-carnitine can reduce the availability of free fatty acids (FFAs), diminish conversion of FFAs to TGs and prevent excess TG accumulation in hepatocytes (32). Interestingly, L-carnitine also can affect cholesterol synthesis pathway (mevalonate pathway) via inhibition of β-hydroxy β-methylglutaryl (HMG)-CoA reductase activity (33, 34). In addition to the benefits of lipids control by L-carnitine in the prevention of CVD, several studies also shown the cardio-protective effects of L-carnitine in terms of reduction of infract size and amelioration of cardiac dysfunction (35). Furthermore, L-carnitine can improve lipid profile by alteration the activity of lipid oxidation enzymes *via* modifying the expression of genes associated with lipid metabolism signaling including peroxisome proliferator activated receptor (PPAR α & γ), and peroxisome proliferator-activated receptor γ coactivator 1α (PGC-1α) (36–39). Also, it is evident that oxidative stress and inflammation can trigger initiation of hyperlipidemia in animals and humans. L-carnitine with anti-oxidant and anti-inflammatory properties can modulate dyslipidemia (40–42). It is important to mention that, protection of LDLs from oxidative stress by L-carnitine can be described *via* few mechanisms, including: oxygen concentration reduced due to enhancement of the β-oxidation of long chain-Acyl CoA (by cause of a large amount of oxygen consumption rates in β-oxidation) and consequently reactive oxygen species formation is decreased (43). Also, L-carnitine can inhibit the activity of enzymes involved in free radical generation and by induction of antioxidant mechanisms (44). Moreover, L-carnitine has been indicated that has a potent for scavenging superoxide anion (45). Additionally, evidence suggests that L-carnitine promotes the synthesis of HDL-C *via* increment in Apo-A1 level (46).

Even though the findings suggest that L-carnitine supplementation may be efficacious for controlling lipid profile, it must be stated that, the effects of L-carnitine on all mentioned lipid parameters were heterogeneous. This heterogeneity may be explained by differences in treatment dosage, gender, mean age, study population, and duration of intervention. Also, the evidence from this study implies that L-carnitine supplementation >2 g/day in subjects with mean age ≤ 50 can meaningfully decrease TC. Similarly, supplementation with L-carnitine in subjects with metabolic disorders with mean age ≤ 50 years and duration of <18-weeks can meaningfully reduce TG levels. In line with TG reduction, L-carnitine intervention in a dose of >2 g/day with duration <18-weeks and with a sample size of >400 participants significantly reduced LDL-C levels, and in people with T2DM, this effect was more significant. Also, L-carnitine consumption had beneficial associations with HDL-C levels in subject with metabolic disorders and intervention duration of <18-weeks.

Aforementioned, the reducing effect of L-carnitine on the level of both TG and LDL-C was shown following short-term supplementation (<18 weeks). Some included studies (19, 30, 47) in our umbrella review indicated that the lowering effect of L-carnitine on TC and LDL-C was achieved when doses of >2 gr/day L-carnitine were consumed. In sum, it seems that the effect of L-carnitine on TG and LDL-C depends on the health condition of individuals, so the patients with metabolic disorders and T2DM have had the most beneficial efficiency of this supplementation.

The results of our investigation indicate that l-carnitine supplementation did not exceed the minimally important difference (MID) in lipid profile (Except LDL-C) in comparison with the control group. The MID concept has been referred to as the minimal clinically important difference or the minimal clinically important improvement. The heterogeneity also in the study characteristics makes it difficult to reach any strong conclusions, particularly in relation to clinical relevance. Therefore, the findings should be considered in the context of these limitations.

Based on pioneering meta-analyses investigating the effect of L-carnitine supplementation on lipid profiles the evidences are contradictory. The main reasons for these discrepancy between the results might partly be due to difference between the included participants, duration of the study, the dosage and types of L-carnitine supplements.

To assimilate the wide number of current evidences available on L-carnitine consumption and dyslipidemia, we done this umbrella review of existing meta-analyses to capture the breadth of outcomes. We found suggestive evidence that L-carnitine may be considered as lipid-modulating agent solo or in concomitant with other lipid lowering drugs. Considerable information from previous studies the L-carnitine was well tolerated without any serious adverse events. However, some trials have reported muscle cramps, asthenia, diarrhea, flu syndrome and headache following high dose (5 g/day or more) of L-carnitine supplementation (19, 23, 30, 48).

The present umbrella meta-analysis study has several strengths. First, this is the first umbrella review to assess the effect of L-carnitine supplementation on lipid profiles with up-to-date literature search strategy from a large number of databases. Second, our umbrella review was registered in the PROSPERO. There were some limitations in our study. First, significant between-studies heterogeneity observed. Second, participants of included studies were from people with different health statuses that leads to indirectness. However, we performed subgroup analysis to present a comprehensive view on the anti-hyperlipidemia effectiveness of L-carnitine.

## Conclusion

In conclusion, although the results indicated that L-carnitine supplementation resulted in a clear improvement in lipid profile in terms of reduction in TC, LDL-C and TG, and significant increasement in HDL-C levels, nevertheless, further large scale RCTs are needed in order to receive a definite conclusion.

## Data availability statement

The original contributions presented in the study are included in the article/Supplementary material, further inquiries can be directed to the corresponding author.

## Author contributions

NR and VM designed research. ZK, MK, and HA conducted research. VM performed statistical analysis. ZK, MK, PD, and EM wrote paper. VM and NR had primary responsibility for final content. All authors contributed to the article and approved the submitted version.

## Conflict of interest

The authors declare that the research was conducted in the absence of any commercial or financial relationships that could be construed as a potential conflict of interest.

## Publisher’s note

All claims expressed in this article are solely those of the authors and do not necessarily represent those of their affiliated organizations, or those of the publisher, the editors and the reviewers. Any product that may be evaluated in this article, or claim that may be made by its manufacturer, is not guaranteed or endorsed by the publisher.
