# Supplementary material for: The effect of L-carnitine supplementation on lipid profile in adults: an umbrella meta-analysis on interventional meta-analyses
Source: Front Nutr. 2023 Sep 4;10:1214734. doi: 10.3389/fnut.2023.1214734 (PMC10506516; doi:10.3389/fnut.2023.1214734)
Supplement: Supplementary file 1 [file Data_Sheet_1.docx]

**TG**
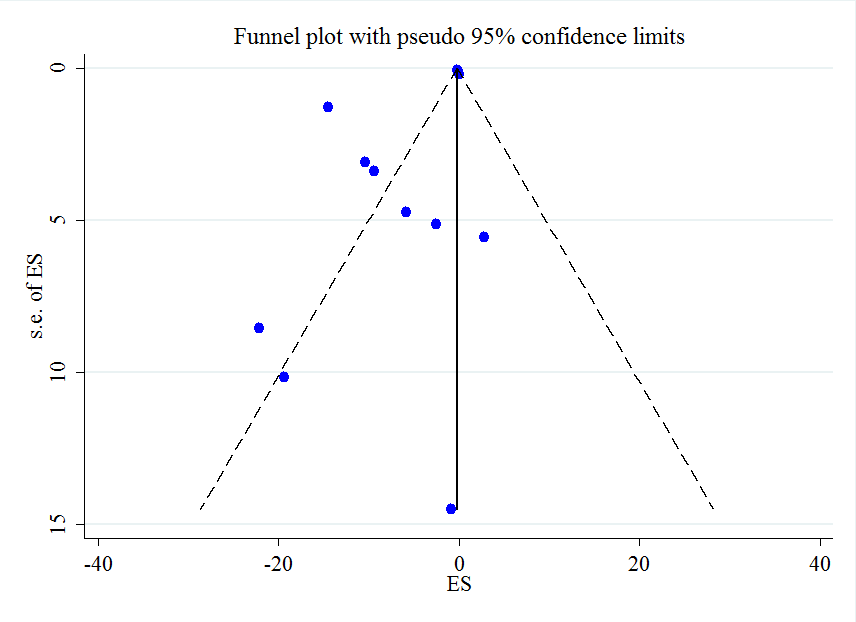


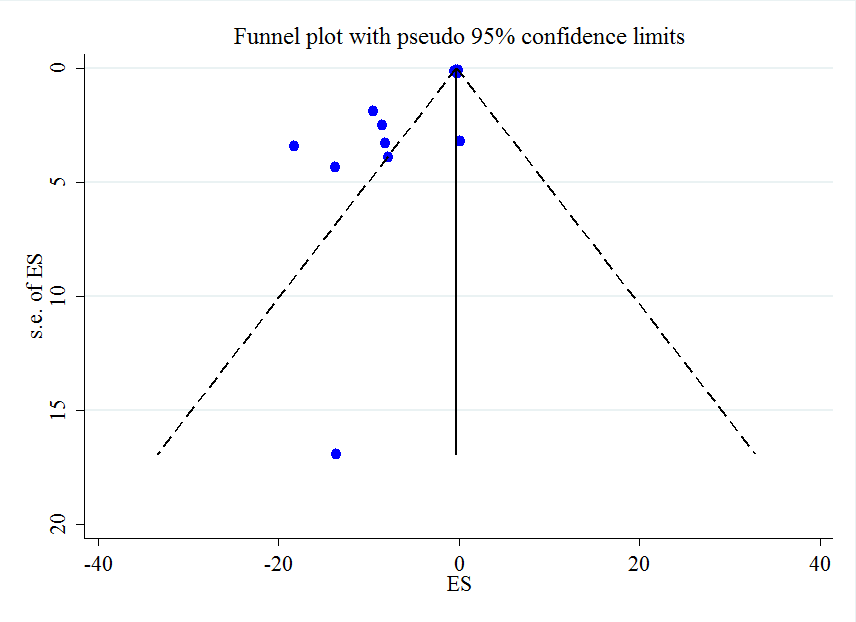


**TC**

**LDL-C**
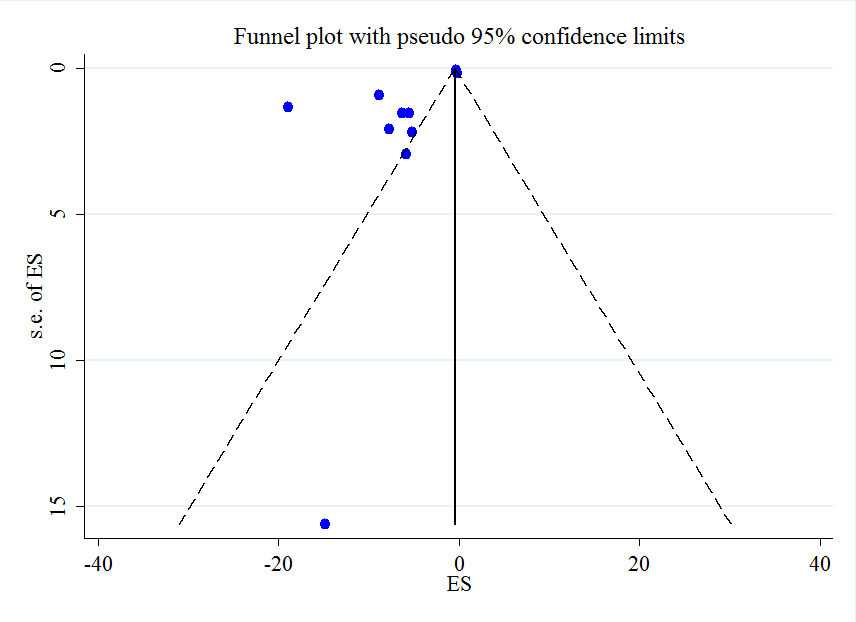

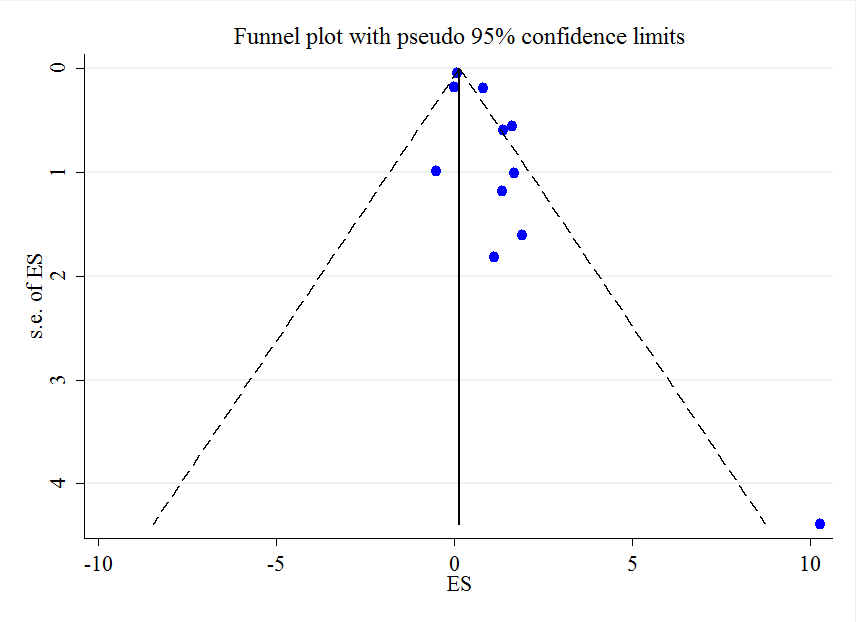


**HDL-C**

Based on MeSH and text keywords, the following pattern of search was applied:

[“Carnitine” [Mesh] OR “Acetylcarnitine”[ Mesh] OR “L-carnitine”[tiab] OR “carnitine”[tiab] OR “levo-carnitine”[tiab] OR “acetyl-L-carnitine”[tiab]" OR “ACAL”[tiab]" **AND** "cholesterol"[Mesh] OR lipids [Mesh] OR Total cholesterol [tiab] OR TC [tiab] OR Triglyceride [tiab]OR TG [tiab] OR High density lipoprotein cholesterol [tiab] OR HDL-C [tiab] OR " Low density lipoprotein cholesterol "[tiab] OR “LDL-C”[tiab] **AND** “systematic review” [tiab] OR “meta-analysis” [tiab]].
